# Role of Dardarin and Isthmin-1 in the Protective Effect of Hydroxytyrosol Against Corn Syrup-Induced Liver Damage

**DOI:** 10.7759/cureus.76803

**Published:** 2025-01-02

**Authors:** Elif Onat, Nevin Kocaman, Serhat Hançer, Murat Yildirim

**Affiliations:** 1 Department of Medical Pharmacology, Fırat University, Elazığ, TUR; 2 Department of Histology and Embryology, Fırat University, Elazığ, TUR; 3 Department of Pediatrics, University of Wisconsin-Madison, Madison, USA

**Keywords:** corn syrup, dardarin, hydroxytyrosol, isthmin-1, liver

## Abstract

Introduction: In this study, it was investigated whether dardarin (LRRK2) and isthmin-1 (ISM1) play a role in the protective effect of hydroxytyrosol (HT) used to prevent liver damage caused by corn syrup in rats.

Methods: Rats were divided into four groups with six in each group: 1) control, 2) HT, 3) corn syrup, and 4) corn syrup + HT. Rats were given water containing 30% corn syrup for six weeks. At the same time, HT-containing liquid was given orally at 4 ml/kg/day, alone and together with corn syrup for six weeks. The weights of the rats were measured every week. LRRK2 and ISM1 molecules in liver tissue were evaluated by histopathological methods. Biochemical parameters were also examined with the enzyme-linked immunosorbent assay (ELISA) method.

Results: It was found that weight gain was less in rats receiving HT than in those consuming corn syrup. The increase in cholesterol, triglyceride, and liver enzyme levels because of corn syrup consumption decreased with HT consumption. As a result of histopathological analysis, it was observed that the increase in LRRK2 and ISM1 levels observed in the liver tissue in the corn syrup-administered group decreased when HT was administered together with corn syrup. In addition, it was determined that the increase in sinusoidal expansion and hepatocyte necrosis observed in the liver tissue as a result of corn syrup application decreased as a result of the application of HT together with corn syrup.

Conclusion: The protective effect of HT against the damage caused by corn syrup in the liver has been demonstrated once again; however, LRRK2 and ISM1 are thought to contribute to this issue.

## Introduction

Excessive consumption of refined sugars, especially high-fructose corn syrup (HFCS), is recognized as among the major contributing factors to the increasing incidence of lifestyle diseases, which include type 2 diabetes mellitus (T2DM), metabolic dysfunction-associated steatotic liver disease (MASLD) and its aggressive form, non-alcoholic steatohepatitis (NASH), some cancers, especially liver, pancreatic, and colon cancers, and cardiovascular and kidney diseases [1]. HFCS has been banned in many countries but is still found in approximately 40% of sweeteners in the United States [1]. HFCS, which is semi-artificial, contains approximately 25-50% more fructose than glucose [2].

Olive leaf extracts have always attracted the attention of many previous researchers to date because of their phenolic components, which are associated with strong biological activities [3]. Hydroxytyrosol (HT), which is one of these, was shown to have antiatherogenic, antiproliferative, and antidiabetic characteristics [4,5]. In vitro studies report that HT can improve endothelial dysfunction, lipid, and hemostatic disorders due to its anti-inflammatory effect along with its antioxidant properties [6]. Also, many in vivo studies report the protective effect of HT on body weight gain and metabolic damage in a rat obesity model [7]. However, the mechanisms underlying these protective characteristics of HT have not yet been elucidated completely.

As a large multidomain protein of 286 kDa, dardarin (LRRK2) belongs to the Roco protein family [8]. Previous cell-related biological studies report that LRRK2 is effective in neurite outgrowth, mitochondria functions, cell repair, synthesis and transport of vesicles, regulation of the endolysosomal system, autophagy and cell death, immune system, protein translation, neurotransmitter, and activity of the intestinal network [9]. Although LRRK2 is attributed to a wide variety of biological processes, its precise functional role has not yet been clearly understood.

Isthmin-1 (ISM1) is a newly identified protein. It has been reported to be expressed in many tissues and organs and to be organ- and tissue-specific in adulthood [10]. The expression of ISM1 in different regions in different animals suggests that ISM1 has various functions. ISM1 was discovered to be expressed in mature adipocytes and stimulate glucose uptake independently of insulin, inhibit hepatic fat synthesis, and stimulate hepatic protein synthesis [11]. It is thought that these findings may create new targets for the prevention and treatment of diseases related to disorders in glycolipid metabolism, such as T2DM and MASLD [12].

The protective effects of HT in metabolic disorders are partially known. Therefore, the protective effect of HT against liver damage caused by consuming corn syrup in rats and the role of proteins such as LRRK2 and ISM1, which are current therapeutic targets for the prevention of many pathologies (neurological, cardiovascular, endocrine, metabolic diseases, and cancer), were investigated in the present study.

## Materials and methods

Animals and experimental design

The study protocol was approved by the Animal Ethics Committee of Adıyaman University on February 8, 2024 (protocol no: 2024/002). The experiments were carried out in accordance with the "Guide for the Care and Use of Laboratory Animals." The study duration was determined as six weeks [13]. Twenty-four male Sprague-Dawley rats, 8-10 weeks old and weighing 200-250 g, supplied by Adıyaman University Experimental Research Center, were used in the study. The rats were kept in a constant-conditioned environment and were allowed to consume standard food and water. The rats were divided into four groups (n = 6): 1) control, 2) HT, 3) corn syrup, and 4) corn syrup + HT. No treatment was applied to the control group. The liquid form of HT was supplied by Kale Naturel Herbal Products Company in Turkey. Rats in groups 2 and 4 were given HT orally at 4 ml/kg/day for six weeks [13]. At the same time, 30% corn syrup was added to the drinking water of rats in groups 3 and 4 for six weeks [14]. At the end of six weeks, rats were anesthetized with intraperitoneal ketamine (75 mg/kg) + xylazine (10 mg/kg), and blood samples were taken from the hearts of the study groups. Liver tissues were taken for fixation in 10% formaldehyde solution for immunohistochemical examination.

Serological analyses

Cardiac blood samples of the nonfasted rats were centrifuged at 4℃ and 10,000 g for 30 minutes. Serum samples were immediately stored at -80℃ until the samples were assayed. Total cholesterol, triglyceride, high-density lipoprotein (HDL), low-density lipoprotein (LDL), aspartate aminotransferase (AST), and alanine aminotransferase (ALT) levels were determined by standard enzymatic techniques.

Histochemical examination

Liver tissues were embedded in paraffin blocks by applying histological follow-up protocol. About 5 µm thick sections were taken from the blocks. Then hematoxylin and eosin, Masson trichrome, and immunohistochemical stains were applied.

Immunohistochemical examination

Immunohistochemical procedures were applied to sections taken from blocks of liver tissues [15]. Immunohistochemistry (IHC) was performed using 3 µm thick histological tissue microarray slides. LRRK2 primary antibody (orb500678; Biorbyt Ltd., Cambridge, UK) and ISM1 primary antibodies (E-AB-18133, Elabscience Biotechnology Inc, Texas, USA) were used. Evaluation and documentation were performed on a Zeiss Axio Scope A1 microscope (Carl Zeiss Microscopy GmbH 07745 Jena, Germany). At the end of the immunohistochemical staining series, histocores were created for LRRK2 and ISM1.

Microscopic evaluation of staining intensity was categorized as follows: negatively colored areas were given a value of 0, areas showing less than 25% staining were given a value of 0.1, areas showing 26-50% staining were given a value of 0.4, areas showing 51-75% staining were given a value of 0.6, and areas showing staining close to homogeneity (76-100%) were given a value of 0.9. The formula used to create histoscores was [15]: \[ \text{Histoscore} = \text{Distribution} \times \text{Intensity} \]

Statistical analysis

IBM SPSS Statistics for Windows, Version 22 (Released 2013; IBM Corp., Armonk, New York, United States) was used for statistical evaluation. A one-way ANOVA test was performed. Tukey HSD tests were performed for post-hoc multiple comparisons. The results were expressed as mean ± standard deviation (SD) with a significance level of p < 0.05 indicating statistical significance.

## Results

Weight values

It was observed that the weight gain in rats was lower as a result of HT administration from the fourth week compared to the control group (p < 0.05). It was observed that the weight gain increased more significantly in the group given corn syrup from the second week compared to the control group and that the weight gain was less as a result of HT administration together with corn syrup after the second week compared to the control (p < 0.05). It was observed that the weight gain was lower as a result of HT administration together with corn syrup from the first week compared to the group given only corn syrup (p < 0.001) (Table 1).

**Table 1:** Weights of the rats ^a^p < 0.05 compared to control; ^b^p < 0.05 compared to HT; ^c^p < 0.05 compared to corn syrup One-way ANOVA test was used. Tukey HSD test was applied for post-hoc multiple comparisons. Data are expressed as mean ± SD (x ± y) with the significance level set at p < 0.05 indicating statistical significance.

Groups	Control	HT	Corn syrup	Corn syrup + HT	p-value
1st day	290.67 ± 27.14	307.83 ± 50.96	295.17 ± 29.43	274.33 ± 58.55	
1st week	320.67 ± 27.15	315.83 ± 49.84	356.67 ± 12.52	275.83 ± 35.89^c^	0.006
2nd week	350 ± 27.96	324.50 ± 49.16	423.67 ± 17.78^a, b^	277 ± 37.38^a, c^	<0.05
3rd week	380 ± 27.97	361.67 ± 64	459.17 ± 29.12^a, b^	275.83 ± 43.88^a, b, c^	<0.05
4th week	400 ± 27.95	316.67±56.87^a^	445.67 ± 29.97^b^	286.33 ± 50.08^a, c^	<0.05
5th week	423.33 ± 26.01	348.17 ± 51.49^a^	468 ± 30.70^b^	296.67 ± 49.73^a, c^	<0.05
6th week	451.50 ± 26.22	363.33± 50.49^a^	497.83 ± 36.99^b^	289.67 ± 39.76^a, b, c^	<0.001

Biochemical findings

As seen in Table 2, plasma total cholesterol, triglyceride, AST, and ALT levels were significantly increased in rats given corn syrup compared to the control group (p < 0.001). Plasma LDL values ​​were higher in rats given corn syrup (p = 0.033). Plasma total cholesterol, triglyceride, AST, and ALT values ​​were lower in the group given HT together with corn syrup than in the group given only corn syrup (p < 0.001). In addition, plasma HDL values ​​were higher in the group given HT together with corn syrup than in the group given only corn syrup (p = 0.003).

**Table 2:** Levels of various biochemical parameters in blood serum of rats ^a^p < 0.05 compared to control; ^b^p < 0.05 compared to HT; ^c^p < 0.05 compared to corn syrup One-way ANOVA test was used. Tukey HSD test was applied for post-hoc multiple comparisons. Data are expressed as mean ± SD (x ± y) with the significance level set at p < 0.05 indicating statistical significance. HDL: high-density lipoprotein; LDL: low-density lipoprotein; AST: aspartate aminotransferase; ALT: alanine aminotransferase

Groups	Control	HT	Corn syrup	Corn syrup + HT	p-value
Total cholesterol (mg/dl)	61.67 ± 4.08	62.67 ± 8.41	85.17 ± 7.05^a, b^	68 ± 2.45^c^	0.001
Triglyceride (mg/dl)	47.5 ± 5.24	50 ± 18.62	111.17 ± 7.55^a, b^	48.33 ± 15.34^c^	<0.001
HDL (mg/dl)	45 ± 5.48	49.33 ± 8.12	50.67 ± 3.88	68.5 ± 8.92^a, b, c^	<0.05
LDL (mg/dl)	5.17 ± 0.41	6.1 ± 2.98	8.17 ± 0.98^a^	7.17 ± 0.41	0.33
AST (U/L)	56.67 ± 5.16	105.50 ± 2.74^a^	158.67 ± 16.5^a, b^	89.33 ± 12.09^a, c^	<0.001
ALT (U/L)	25.83 ± 2.04	30.33 ± 4.63	53.5 0 ± 8.60^a, b^	26.83 ± 2.71^c^	<0.001

Histochemical findings

**Figure 1:**
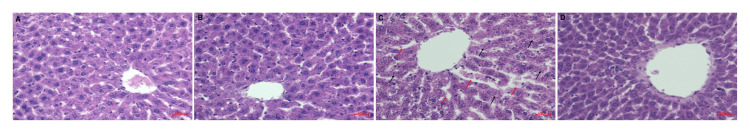
Sinusoidal dilation (red arrow) and hepatocyte necrosis (black arrow) were observed in the liver tissue stained with hematoxylin and eosin A) Control; B) HT; C) Corn Syrup; D) Corn Syrup + HT Scale bar: 100 µm HT: hydroxytyrosol

**Figure 2:**
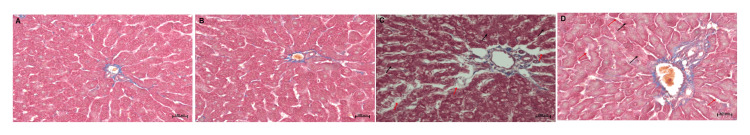
Sinusoidal dilation (red arrow) and hepatocyte necrosis (black arrow) were observed in the liver tissue stained with Masson's trichrome A) Control; B) HT; C) Corn Syrup; D) Corn Syrup + HT Scale bar: 100 µm HT: hydroxytyrosol

As a result of the examination of hematoxylin-eosin and Masson trichrome-stained preparations under the light microscope, the control and HT groups appeared normal (Table 3, Figures 1-2).

**Table 3:** Histopathologic findings of the liver tissues (hematoxylin and eosin and Masson trichrome) ^a^p < 0.05 compared to control; ^b^p < 0.05 compared to HT; ^c^p < 0.05 compared to corn syrup One-way ANOVA test was used. Tukey HSD test was applied for post-hoc multiple comparisons. Data are expressed as mean ± SD (x ± y) with the significance level set at p < 0.05 indicating statistical significance.

Parameters	Control	HT	Corn syrup	Corn syrup + HT	p-value
Sinusoidal dilation	1.29 ± 0.49	1.14 ± 0.38	6.29 ± 0.76^a, b^	1.43 ± 0.53^c^	<0.001
Hepatocyte necrosis	1.57 ± 0.53	1.43 ± 0.53	6.71 ± 0.49^a, b^	4 ± 0.58^a, b, c^	<0.001
Fibrosis	1.71 ± 0.49	1.71 ± 0.49	1.86 ± 0.69	1.86 ± 0.38	

As a result of corn syrup application, sinusoidal dilatation (red arrow) and hepatocyte necrosis (black arrow) increased compared to control and HT groups (p < 0.001). Compared to the corn syrup group, sinusoidal dilatation (red arrow) and hepatocyte necrosis (black arrow) decreased significantly in the corn syrup and HT group (p < 0.001). An insignificant increase in fibrosis was observed in the corn syrup group compared to the control and HT groups. However, fibrosis did not change with corn syrup + HT application (Table 3, Figures 1-2).

Immunohistochemical findings

**Figure 3:**
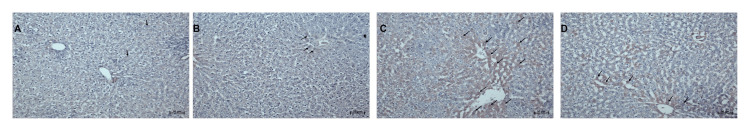
Immunohistochemical findings for dardarin (black arrow) in the liver tissues A) Control; B) HT; C) Corn Syrup; D) Corn Syrup + HT HT: hydroxytyrosol

After examination of liver tissue under a light microscope for immunohistochemical staining for LRRK2 and ISM1 immunoreactivity, LRRK2 immunoreactivity was significantly increased in the corn syrup group compared to the control and HT groups (p < 0.001). In contrast, LRRK2 immunoreactivity was found to be lower in rats given HT with corn syrup compared to those given only corn syrup (p < 0.001) (Table 4). Histocores showing LRRK2 immunoreactivity for all groups are shown in Figure 3. LRRK2 expressions in all groups are shown with black arrows.

**Table 4:** Immunohistochemical findings for dardarin in the liver tissues ^a^p < 0.05 compared to control; ^b^p < 0.05 compared to HT; ^c^p < 0.05 compared to corn syrup. One-way ANOVA test was used. Tukey HSD test was applied for post-hoc multiple comparisons. Data are expressed as mean ± SD (x ± y) with the significance level set at p < 0.05 indicating statistical significance.

Groups	Control	HT	Corn syrup	Corn syrup + HT	p-value
Dardarin	0.35 ± 0.1	0.33 ± 0.09	0.71 ± 0.19^a, b^	0.34 ± 0.07^c^	<0.001

**Figure 4:**
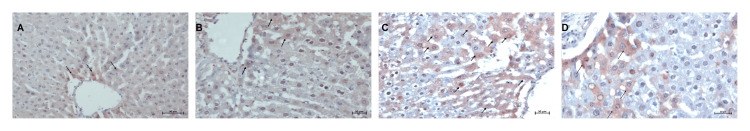
Immunohistochemical findings for isthmin-1 (black arrow) in the liver tissues A) Control; B) HT; C) Corn Syrup; D) Corn Syrup + HT HT: hydroxytyrosol

In terms of ISM1 immunoreactivity, an increase was seen in the given-only corn syrup rats compared to the control and HT groups (p < 0.001). ISM1 immunoreactivity was lower in rats given HT with corn syrup compared to those given only corn syrup (p = 0.005) (Table 5). Histobodies showing ISM1 immunoreactivity in all groups are shown in Figure 4. ISM1 expressions in all groups are shown with black arrows.

**Table 5:** Immunohistochemical findings for isthmin-1 in the liver tissues ^a^p < 0.05 compared to control; ^b^p < 0.05 compared to HT; ^c^p < 0.05 compared to corn syrup. One-way ANOVA test was used. Tukey HSD test was applied for post-hoc multiple comparisons. Data are expressed as mean ± SD (x ± y) with the significance level set at p < 0.05 indicating statistical significance.

Groups	Control	HT	Corn syrup	Corn syrup + HT	p-value
Isthmin-1	0.24 ± 0.11	0.25 ± 0.11	0.73 ± 0.16^a, b^	0.45 ± 0.12^a, c^	<0.05

## Discussion

The Mediterranean diet and its indispensable part, olive oil, are almost synonymous with a healthy diet and lifestyle today and are highly appreciated. It is known that the Mediterranean diet has many positive effects, such as reducing the likelihood of many chronic diseases and extending human life. Although the polyphenols found in olive oil have minor effects in olive oil, they are of great importance for health, and interest in their biological and therapeutic effects is increasing. In addition to interventional clinical studies, in vitro and in vivo studies are also increasing, revealing many new, previously unknown properties of these compounds and their positive effects on health [16]. This study aimed to examine the protective effect of HT, one of the important polyphenols found in olive oil, against liver damage caused by corn syrup intake in rats and the possible roles of LRRK2 and ISM1 proteins in this. As a result of this study, it was concluded that this phenolic compound has a significant pharmacological effect. However, more research is needed to understand its protective mechanism.

Consumption of HT has recently been found to improve insulin resistance and reduce excess weight by regulating intestinal flora, as it is one of the main components of olive oil and has significant antioxidant, anti-inflammatory, and antimicrobial properties and has been linked to the improvement of MetS and related disorders [17]. Olive leaf extracts containing high amounts of HT have lipid-lowering and liver-protective effects on liver damage due to excessive fat consumption and liver damage in rats following the improvement in the antioxidative system and inhibition of the protein expression in inflammation and liver damage against metabolic damage caused by excessive fructose consumption [18]. In the present study, it was shown once again that increased total cholesterol, triglyceride, LDL, AST, and ALT values and weight of rats based on corn syrup consumption decreased with the administration of HT, which has hypolipidemic and hepatoprotective effects, and increased HDL value shows that HT has protective effects on metabolic disorders and obesity. In addition, it was observed that the increase in sinusoidal expansion and hepatocyte necrosis observed in the liver tissue as a result of corn syrup application was significantly reduced as a result of HT application.

As a result of genetic studies, LRRK2 is thought to be associated with Parkinson's disease, Crohn's disease, multibacillary leprosy, and cancer [19]. LRRK2 knockout (KO) mice showed kidney, lung, and liver abnormalities, including vesicle accumulation within cells and autophagic changes [20]. A recent study found that alterations in LRRK2 can cause defective chaperone-mediated autophagy (CMA) [21]. LRRK2 has been reported to interact with various cellular proteins such as β-tubulin, moesin, and actin; this suggests that LRRK2 may play a role in regulating cell structure shaping and neurite outgrowth [22]. Many studies report that LRRK2 functions in tissues containing membranous structures throughout the body [23]. LRRK2 seems to play important roles in various cellular processes that involve membranous structures. However, the connection between lysosomes and LRRK2 has recently attracted attention because some studies show lysosomal abnormalities in Lrrk2-KO animals, such as the accumulation of autofluorescent lipofuscin granules produced in lysosomes [24-26]. In fact, detailed histopathological analyses show a significant expansion of lysosomes and lysosome-associated structures in the kidneys or lungs of Lrrk2 KO rodents [24,25]. Administration of LRRK2 kinase inhibitors to primates also induced abnormal cytoplasmic accumulation of lamellar bodies in type II pneumocytes in the lungs [25]. Therefore, the main function of LRRK2 is thought to be the maintenance of lysosome structure and function [27]. This is further demonstrated by findings from several studies showing aging-related abnormalities in homozygous LRRK2KO rats, progressing to increased vacuolation in the kidneys, lungs, and liver [24]. Studies conducted in homozygous LRRK2 KO mice models reported that there is an age-related increase in renal atrophy and degeneration, abnormal accumulation of α-synuclein and ubiquitinated protein, and disruption in the autophagy-lysosomal pathway, but no neurodegeneration was reported in this respect [28]. Studies with LRRK2 KO animals suggest that LRRK2 is a regulator of colitis, susceptibility to lung cancer, and susceptibility to neurodegeneration [9]. Despite these studies, the exact function and mechanism of LRRK2 remain unknown. In the present study, the fact that LRRK2 protein increased in the liver tissues of the rats given corn syrup brings to our mind the idea that LRRK2 protein might have increased to have a regulatory effect on corn syrup-induced liver damage. The fact that LRRK2 levels were low in the group given HT along with corn syrup since there was no obvious pathology that would require an increase in the amount of LRRK2 protein and because HT has protective effects on liver damage supports our opinion. More studies are needed to determine whether the LRRK2 protein plays a role in the protective effect mechanism of HT.

Recent studies identify ISM1 as an adipokine that stimulates glucose uptake, inhibits lipid synthesis, stimulates protein synthesis, and is associated with obesity in adipocytes and female plasma [11]. Additionally, the amount of ISM1 in the bloodstream has been found to be lower in obese individuals with T2DM than in obese individuals without T2DM; this supports the idea that high ISM1 levels may reduce the likelihood of developing diabetes [29]. ISM1 has been found to be at particularly high levels in brown adipocytes. ISM1, secreted by adipocytes in metabolism, exerts an autocrine effect to regulate glucose uptake via mTORC2-PI3K-AKT together with insulin-IR/IGF-1R-PI3K-AKT. ISM1 can cause ERK phosphorylation [11]. Since ISM1 is similar to insulin in terms of its function and the signaling pathways it affects, it acts like insulin in the regulation of glucose and lipogenesis. However, ISM1 inhibits insulin-dependent expression of the prolipid synthesis factor sterol regulatory element binding protein-1 (cSrebp1c) and its target genes acetyl CoA carboxylase (ACC), fatty acid synthase (FAS), and low-density lipoprotein receptor (LDLR). In this way, it reduces insulin-stimulated lipogenesis [11]. It also affects lipid synthesis by inhibiting the expression of ISM1, Srebp1c, and their target genes in liver cells. It regulates protein synthesis through AKT-mTORC1-S6 in liver and skeletal muscle cells. ISM1 inhibits lipid synthesis by switching the anabolic state in favor of protein synthesis [11]. ISM1 may improve metabolic diseases by reducing oxidative stress and lipid peroxidation because oxidative stress and lipid peroxidation are closely related to diabetes, obesity, and MASLD [30]. The increased liver ISM1 levels in rats because of corn syrup consumption in the present study once again indicate the protective roles of ISM1 in this pathology because ISM1 has the ability to play roles in metabolic diseases by improving inflammation, metabolic disorders, and endoplasmic reticulum stress [12]. Also, the fact that ISM1 levels were low in rats given HT together with corn syrup suggests that ISM1 might have active roles in the protective mechanism of HT. Because HT exerts its protective effects on the liver over (i) regulation of A-5 and A-6 desaturase activity by preventing the reduction of n-3 LCPUFAs, (ii) inhibition of oxidative stress, (iii) lipogenic factor SREBP 1c, and (iv) protection of n-3 LCPUFA levels outside the liver [31]. The fact that some of these action mechanisms of HT are common with ISM1 supports this idea of ours. It is a fact that this information will make a significant contribution to guiding future studies.

Limitations

One of the limitations of this study is that genetic analyses were not performed. The related pathways must be interrogated in studies with larger numbers of animals to provide clear information about the relationship of HT with LRRK2 and ISM1. Also, supporting the protective characteristics of HT with clinical observations will increase the reliability of this component.

## Conclusions

As a result of the present study, it is considered that HT might have protective effects on metabolic disorders such as liver damage, hyperlipidemia, and weight gain caused by corn syrup consumption, and newly discovered proteins such as LRRK2 and ISM1 may also play roles in the mechanisms underlying this effect.
